# Cryptosporidiosis: A Disease of Tropical and Remote Areas in Australia

**DOI:** 10.1371/journal.pntd.0004078

**Published:** 2015-09-22

**Authors:** Aparna Lal, Lisa Michelle Cornish, Emily Fearnley, Kathryn Glass, Martyn Kirk

**Affiliations:** 1 National Centre for Epidemiology and Population Health, Research School of Population Health, Australian National University, Canberra, Australian Capital Territory, Australia; 2 National Centre for Geographic & Resource Analysis in Primary Health Care (GRAPHC), Research School of Population Health, Australian National University, Canberra, Australian Capital Territory, Australia; New York University, UNITED STATES

## Abstract

Cryptosporidiosis causes gastroenteritis and is transmitted to humans via contaminated water and food, and contact with infected animals and people. We analyse long-term cryptosporidiosis patterns across Australia (2001–2012) and review published Australian studies and jurisdictional health bulletins to identify high risk populations and potential risk factors for disease. Using national data on reported cryptosporidiosis, the average annual rate of reported illness was 12.8 cases per 100 000 population, with cycles of high and low reporting years. Reports of illness peak in summer, similar to other infectious gastrointestinal diseases. States with high livestock densities like New South Wales and Queensland also record a spring peak in illnesses. Children aged less than four years have the highest rates of disease, along with adult females. Rates of reported cryptosporidiosis are highest in the warmer, remote regions and in Aboriginal and Torres Strait Islander populations. Our review of 34 published studies and seven health department reports on cryptosporidiosis in Australia highlights a lack of long term, non-outbreak studies in these regions and populations, with an emphasis on outbreaks and risk factors in urban areas. The high disease rates in remote, tropical and subtropical areas and in Aboriginal and Torres Strait Islander populations underscore the need to develop interventions that target the sources of infection, seasonal exposures and risk factors for cryptosporidiosis in these settings. Spatial epidemiology can provide an evidence base to identify priorities for intervention to prevent and control cryptosporidiosis in high risk populations.

## Author Summary

The parasite *Cryptosporidium* is a common cause of gastroenteritis worldwide. Ineffectively focused interventions are partly why the disease remains a challenge to control. In this study, we describe the geographical, seasonal and demographic characteristics of reported cryptosporidiosis in Australia from 2001–2012. We combine this analysis of illnesses with evidence published in peer review articles and state health bulletins to identify high disease risk areas and populations. We find that rates of reported cryptosporidiosis are highest in warm, remote areas and in Aboriginal and Torres Strait Islander populations’ dominated regions. Our review of the published literature and health reports highlights a focus on short term outbreaks in metropolitan areas. This negligible overlap between areas with high disease rates and areas of public health focus is of concern. Public health interventions and promotion programs to prevent and control diarrhoea need to focus on remote and Indigenous dominated Australia to reduce the currently high rates in these regions and populations.

## Introduction

Cryptosporidiosis, caused by the intestinal parasite *Cryptosporidium*, is an important cause of gastroenteritis worldwide, particularly in resource limited settings [1]. Infection commonly presents as self-limiting gastroenteritis, but in the immune-compromised, children and the elderly can result in persistent infection, malnutrition and, more rarely, death [2–4]. *Cryptosporidium* is transmitted via the faecal-oral route and is easily spread through water [5], food [6], contact with infected animals [7,8], contaminated environments [9] and through contact with infected persons [10]. The pathogen requires a low infectious dose, oocysts are immediately infectious once excreted and are resistant to traditional water treatment methods for drinking water supplies and swimming pools, such as chlorination [11–13]. These characteristics make *Cryptosporidium* ubiquitous in the environment and the disease a challenge to control. Despite its importance worldwide, our limited understanding of the sources of infection and pathways for spread has resulted in ineffective public health strategies to prevent human infection [14,15].

Cryptosporidiosis is recognized as a parasitic disease with suboptimal disease prevention measures resulting in high disease rates in some populations in Oceania, similar to other places [16]. Identification of areas and time periods with consistently high rates of infection can help determine the environmental, socio-economic and demographic risk factors for disease. This knowledge can inform the development of targeted public health interventions to reduce disease. To date, there are no national descriptions of the seasonal, spatial and population-specific patterns of reported cryptosporidiosis in Australia.

Cryptosporidiosis became a nationally notifiable disease in Australia in 2001 [17]. We use Geographic Information Systems (GIS) approaches to identify and visualize high risk populations across Australia from 2001 to 2012. Our analysis provides an insight into environmental, social and demographic factors at the population level that may result in localized areas and time periods of high cryptosporidiosis risk. Combined with a review of published literature on cryptosporidiosis in Australia, we identify high risk groups that may benefit from targeted interventions for disease control.

## Methods

### Literature review of cryptosporidiosis in Australia

Using three electronic databases, PubMed, Web of Science and Embase, publications that focused on cryptosporidiosis in Australia were identified. The keywords used were: (“Australia”), AND (“cryptosporidiosis”, “cryptosporidium”). No language or database or time restrictions were imposed on the searches. Full-text versions of the articles that focused on cryptosporidiosis in humans were obtained and their reference lists were manually searched to identify any further relevant manuscripts. Bibliographies of reviews published on *Cryptosporidium* epidemiology were also examined to identify additional sources for inclusion in the review. We also searched jurisdictional health departments’ public health bulletins in order to capture *Cryptosporidium* spp. outbreak reports and surveillance summaries. For the relevant studies, the following information was recorded: authors and year of publication, location, outbreak or sporadic, study design, the main risk factors or potential sources of infection identified.

### Data sources

All cases of cryptosporidiosis reported during 2001–2012 in Australia were obtained from the National Notifiable Diseases Surveillance System, which is overseen by the Communicable Disease Network Australia and managed by the Australian Government Department of Health. Case data obtained included notification date, sex, age (in five year age groups), Aboriginal and Torres Strait Islander (ATSI) status, state and postcode of residence. Ethical approval for the study was obtained from the Australian National University prior to data release. For notifications, cases were defined according to the national case definition for confirmed cases, requiring laboratory definitive evidence only with no clinical criteria [17,18].

Annual population denominator data by age, sex, ATSI status and state were based on Estimated Resident Population (ERP) estimates produced annually by the Australian Bureau of Statistics (ABS). Geographical State and Territory and postal area boundaries were obtained from the ABS. The Postal area boundaries produced by the Australian Bureau of Statistics differ between each census, due to population changes, changes to postal distribution areas and changes to the ABS methodology in defining postal area boundaries. For the 2001 and 2006 census, postal area boundaries were based on census districts and for 2011, the methodology was changed to ensure postal areas matched Statistical Area 1 boundaries (a non-administrative, geographical unit defined by the Australian Bureau of Statistics). This resulted in postal areas being removed, added and boundary changes. The ABS has not produced correspondence files allowing postal areas from 2001 and 2006 to be matched to 2011 boundaries. Therefore in this paper, data from 2001–2005 were mapped using the 2001 postal area boundaries; data from 2006–2010 were mapped using the 2006 postal area boundaries and data from 2011–2012 were mapped using the 2011 postal area boundaries. More information on postal area boundaries can be found at www.abs.gov.au.

### Analysis

National, State/Territory, age and sex specific cryptosporidiosis rates were calculated using the annual ERPs for each year from the ABS and converted to rates per 100 000 population. Seasonal patterns were visualized using the total number of notifications by week over the study period for each State and Territory. As data were available at the postal area level, using the ABS index of remoteness for 2011[19], each postcode was assigned a value of “remoteness” to describe cases by location. The ABS classification for remoteness designates each area as either ‘major cities’ ‘inner regional’, ‘outer regional’, ‘remote’ or ‘very remote’. To generate maps of reported illness by ATSI status, the total number of notified ATSI cases in each region was divided by the total ATSI population from the relevant census and converted to rates per 100,000 population. It is important to note that there is variation across the states and territories in the completeness of reporting of ATSI status for cryptosporidiosis. Following the methods of the Australian Institute of Health and Welfare, to assess completeness of reporting by State and Territory, we used a cut-off of 50% completeness of ATSI status for the period 2009–2012 [20]. All States and Territories met this criterion, apart from Victoria (48% complete with 52% of records with a blank field for ATSI status) and Queensland (44% complete with 56% of records with a blank field for ATSI status). Victoria and Queensland were excluded from the calculation and visualization of rates of reported cryptosporidiosis for ATSI populations. Stata v 13.0 and Microsoft Excel 2010 were used for data management and analysis including rate calculations and confidence intervals (using the command “ci” to generate the mean, standard error and 95% confidence intervals for each category). ArcGISv10.0 was used for mapping.

## Results

### Literature review of cryptosporidiosis in Australia

We found 38 relevant published studies on cryptosporidiosis in Australia, of which 34 studies are summarized in S1 Table. The full texts of four studies published prior to 1993 were not available. We found 9 additional reports from searching the jurisdictional health bulletins. Of these, one was published as a peer reviewed research article while two descriptions reported on the same outbreak. Together, there were 41 individual reports on cryptosporidiosis in Australia. Overall, 56% (23/41) of published studies were conducted in urban settings, 14% (6/41) focused on remote regions, 27% (11/41) were state-wide analyses and the locations in which samples were obtained were unknown for three studies. Of the eight studies that used a case-control study design, six identified swimming pools as the main risk factor for outbreaks and sporadic cases in urban areas. Consumption of unpasteurized milk and calf contact at an agricultural show and animal petting farms were identified as risk factors for three other outbreaks. Of the molecular focused studies, 83% (10/12) identified cattle subtypes in human infections, two identified a kangaroo subtype in a human infection and one case report described a wildlife associated genotype. Four studies used data for over five years with data limited to state specific reported incidence.

### Time trends and seasonal patterns

**Fig 1:**
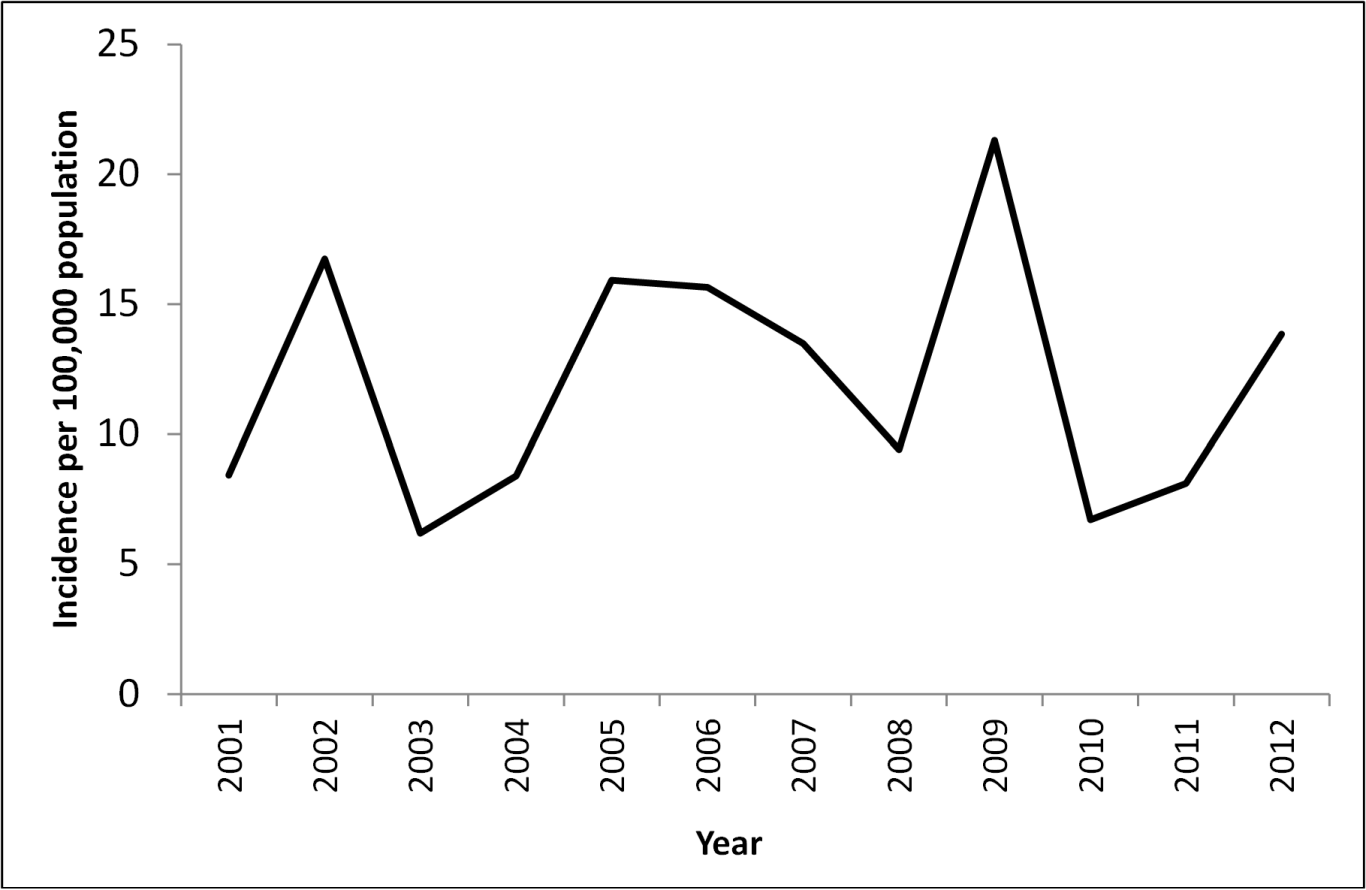
Average annual rates of reported cryptosporidiosis per 100 000 population across Australia, 2001–2012.

The average annual rate of reported cryptosporidiosis was 12.8 cases per 100 000 population. Cryptosporidiosis notifications showed distinct cycles over the 12 year period from 2001 to 2012. The rate of reported illnesses peaked in 2002, 2005 and 2009, and was lower in the years 2003, 2008 and 2010 (Fig 1). The number of illnesses was highest in 2009 with 4625 notified cases nationally.

**Fig 2:**
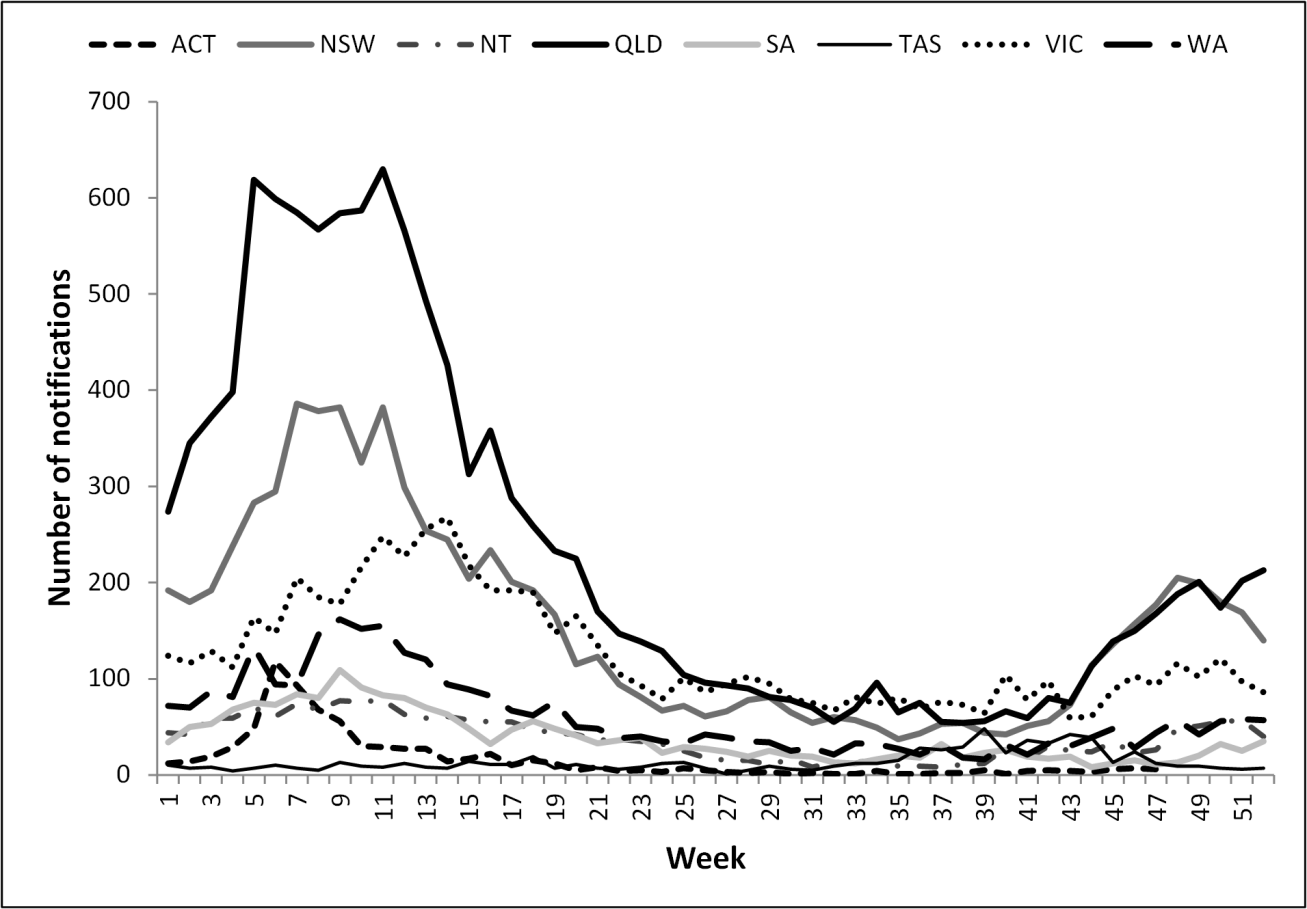
Total weekly number of cryptosporidiosis notifications across Australian states and territories, 2001–2012.

A seasonal pattern of disease notification remained consistent throughout the reporting period and was evident in most States and Territories (Fig 2). Notifications peaked towards the end of summer starting in week six (February) with the Australian Capital Territory, and ending with the Northern Territory in week 9 (early March) (Fig 2). Tasmania, Queensland and New South Wales also saw rising numbers of notifications in spring, from week 39 (September) onwards. Victoria also has a smaller, later spring peak from week 45 (late October) onwards.

### Geographic distribution

**Fig 3:**
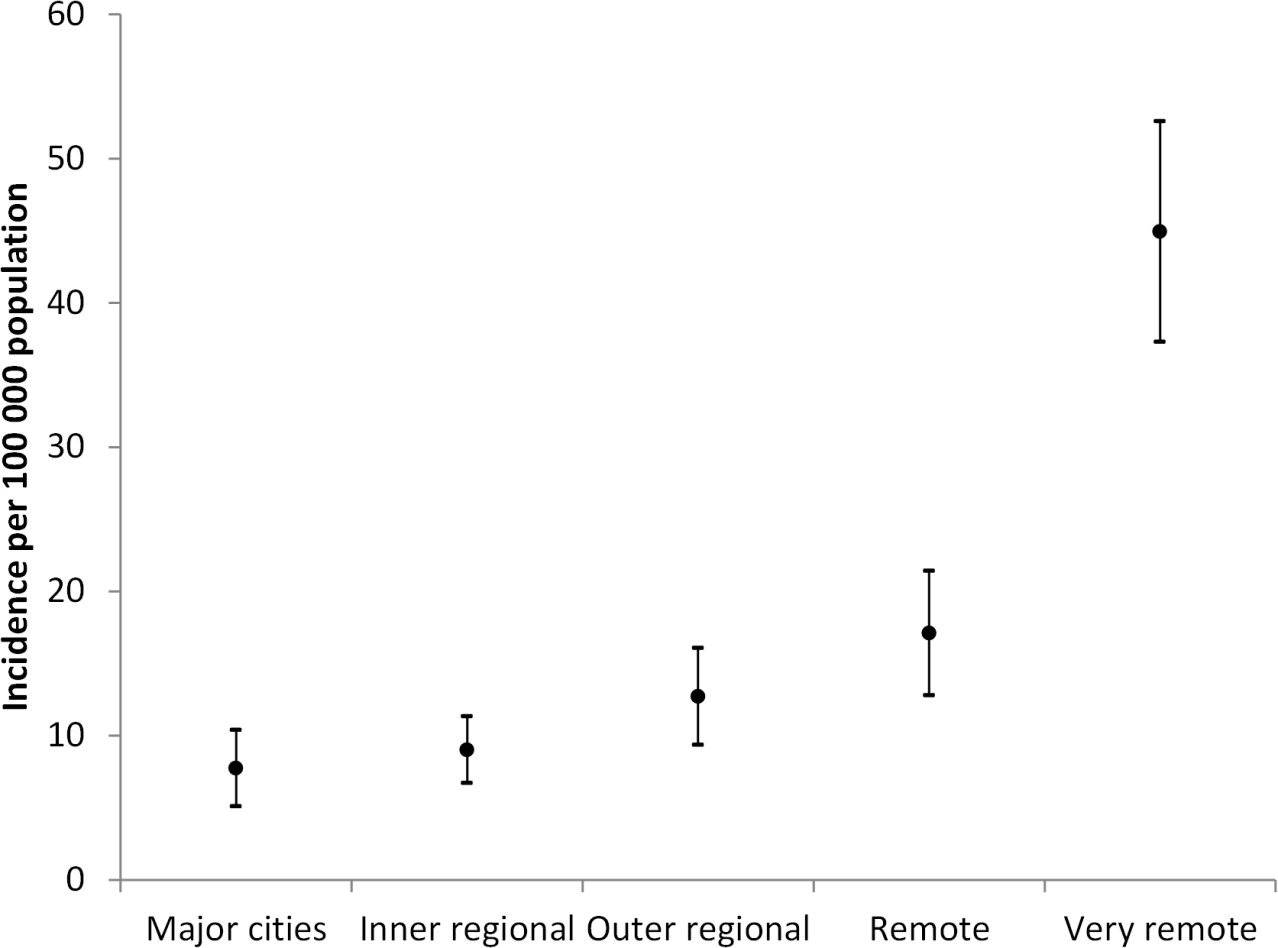
Average rates of reported cryptosporidiosis per 100 000 population (with 95% confidence Intervals) by remoteness category across Australia, 2001–2012.

Average annual cryptosporidiosis rates of reported illness by remoteness category are shown in Fig 3. The lowest rate of notifications was in the major cities (7.7 per 100 000 population), followed by inner regional areas (9.0 per 100 000 population), outer regional areas (12.7 per 100 000 population), and remote areas (17.1 per 100 000 population). The areas categorized as “very remote” had the highest average rates of notification (44.9 per 100 000 population).

### Demographic trends

Overall, the highest number of reported illnesses was seen in children aged 0–4 years, comprising 45% (15852/35455) of all reported illnesses (Table 1). Overall, males and females showed a similar distribution of illnesses with 17551 (49.5%) and 17904 (50.5%) illnesses respectively. Queensland had the highest number of reported illnesses (n = 12271), while Tasmania had the lowest (n = 706) (Table 1). Average annual rates of reported cryptosporidiosis vary by age, gender and geographic region (S1 Fig). Using annual population estimates for each age and gender group, all States and Territories had the highest average notification rates in boys aged 0–4 years and higher rates for adult females across the age groups 20 to 39 years compared to males (S1 Fig).

**Table 1:** Number and percentage of reported cryptosporidiosis by age group, gender and State or Territory of residence, 2001–2012.

Characteristic	Number of illnesses	% of total	Average annual crude rates per 100 000 population
**Age groups (years)**			
0–4	15852	44.7	105.2
5–9	4622	13.0	13.4
10–14	1991	5.6	6.1
15–19	995	2.8	2.6
20–24	1553	4.3	4.4
25–29	2105	5.9	5.1
30–34	2627	7.4	7.0
35–39	2113	5.9	5.5
40–44	1028	2.9	2.8
45–49	583	1.6	2.2
50–54	472	1.3	1.4
55–59	445	1.2	2.1
60–64	321	0.9	2.0
65–69	260	0.7	1.4
70–74	150	0.4	1.1
75–79	132	0.3	1.3
80–84	82	0.2	4.3
85+	84	0.2	3.2
**Gender**			
Male	17551	49.5	9.6
Female	17904	50.4	9.4
**State**			
Queensland	12271	34.6	20.7
New South Wales	8082	22.8	7.9
Victoria	6514	18.3	9.8
Western Australia	3152	8.8	12.4
Northern Territory	1986	5.6	63.3
South Australia	1983	5.5	8.9
Australian Capital Territory	761	2.1	8.1
Tasmania	706	1.9	9.1

**Fig 4:**
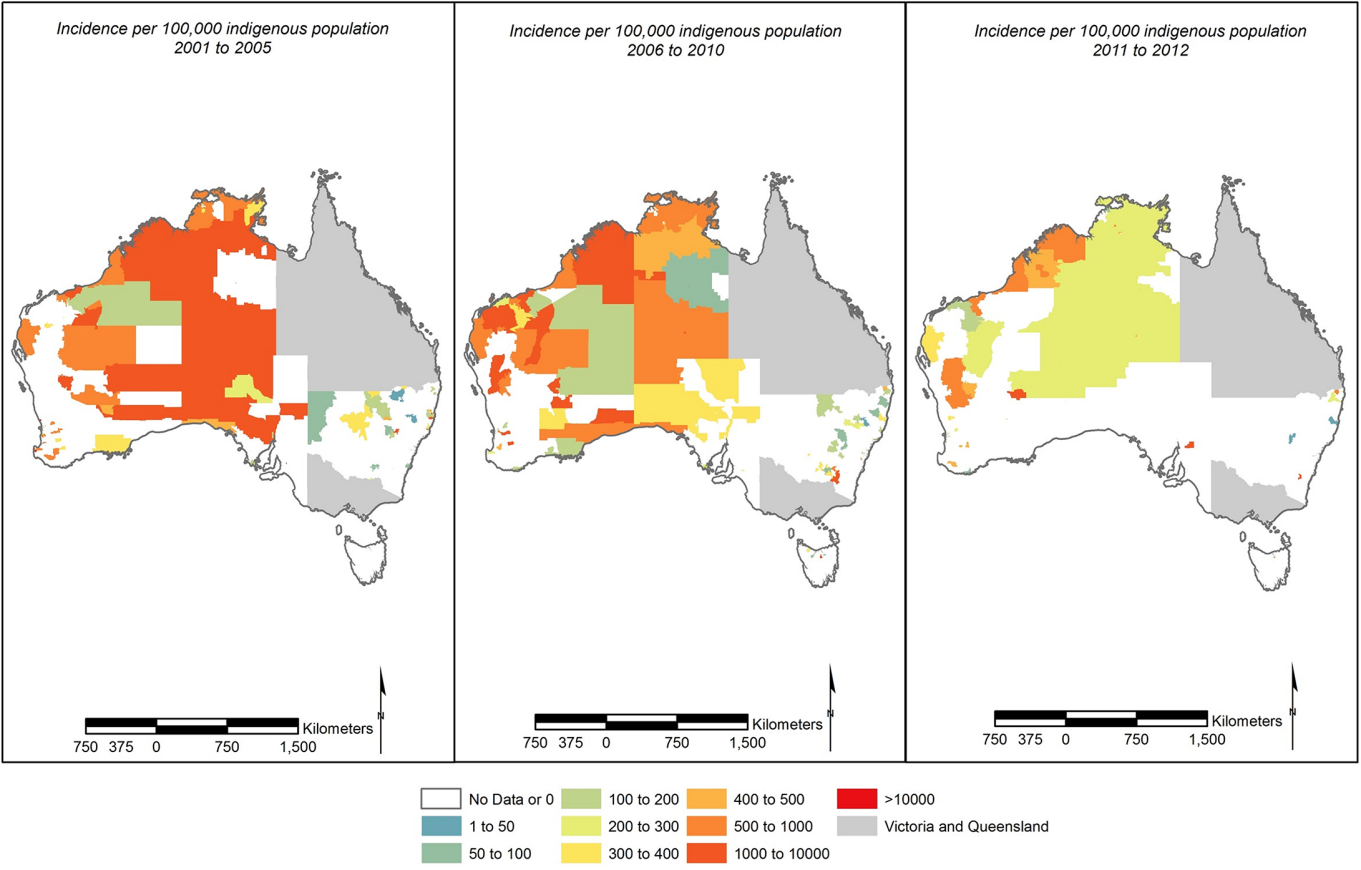
Average rates of reported cryptosporidiosis per 100 000 indigenous population (excluding Queensland and Victoria) for (A) 2001–2005 using 2001 postal area boundaries (B) 2006–2010 using 2006 postal area boundaries (C) 2011–2012 using 2011 postal area boundaries.

Over the 12 year period, rates of reported cryptosporidiosis in ATSI populations were highest in the northern, tropical region of Australia with high rates in parts of Western Australia (Fig 4). When the rates of reported cryptosporidiosis for the general population were viewed in relation to the distribution of climatic regions and remoteness categories identified by the ABS (S2 Fig), we observed consistently high rates of reported cryptosporidiosis in the warmer tropical and subtropical, remote and very remote areas (S3 Fig).

## Discussion

Rates of reported cryptosporidiosis in Australia are 12.8 illnesses per 100 000 population. Rates in other developed countries with similar surveillance systems are generally lower at 2.9 per 100 000 (United States) [21] and 2.7 per 100 000 (Canada) and 8 per 100 000 (England and Wales), but higher in New Zealand at 22 per 100 000 [22]. These high rates support *Cryptosporidium*’s status as an important parasitic infection of the Oceanic region [16]. Cryptosporidiosis patterns in Australia show marked geographic and demographic variations in disease reporting rates. The high rates of reported illness in the warmer, remote and Indigenous dominated areas in Australia combined with the lack of literature on non-outbreak disease patterns in these settings is of concern.

Environmental transmission cycles between humans and animals or contaminated environments are likely to be an important mode of disease spread in some regions. Livestock are an important reservoir of *Cryptosporidium*. Of interest is the increase in the number of reported illnesses in spring in Queensland and New South Wales. According to the 2011 Agricultural Census [23], these States were the leading cattle (dairy and meat) rearing regions in Australia. This seasonal pattern may be due to transmission from young livestock born at this time, as young calves harbor high loads of the *Cryptosporidium* strain known to cause disease in humans [24–26]. Spring peaks in reported cryptosporidiosis in humans have been documented to coincide with the presence of young livestock, notably cattle, known to carry a considerable load of *Cryptosporidium* spp. capable of causing human infection in New Zealand and the UK [8,24,27]. In Australia, associations between cryptosporidiosis and cattle are evident from our review of published studies which include molecular studies indicating the existence of cattle adapted strains in human cases [28–31], outbreaks related to the consumption of unpasteurized milk [32] and attendance at an animal fair [33] and an animal petting farm [34]. Of interest is the increasing molecular evidence of many different strains of *Cryptosporidium* in the environment and animals, whose potential for infection in humans is largely unknown [35].

Strong seasonal patterns in the number of reported illnesses are also evident in other states and territories, with increasing numbers of reported cases in December through to March; a pattern maintained throughout the reporting period. A study included in our review shows that in Brisbane, a subtropical city of Queensland, Australia, hot temperatures and dry conditions were associated with an increased risk of cryptosporidiosis [36]. Globally, cryptosporidiosis shows a strong association with rainfall and temperature variability [37,38] across a wide range of climatic regions and countries [39]. Quantifying the association between disease risk and environmental factors such as weather variability and proximity to livestock and wildlife can help identify populations at imminent risk of infection.

Residing in remote or rural regions has been identified as a risk factor for cryptosporidiosis in studies published elsewhere [40,41]. We show that in Australia, rates of reported cryptosporidiosis increase with increasing remoteness. Rural living as a risk factor for cryptosporidiosis is particularly linked to cattle density [42]. Our review of the published literature suggests that cattle are likely to be an important reservoir of human infection in some rural areas. However, remote living may also be associated with less than ideal hygienic conditions for food preparation and storage [43], poor housing and household overcrowding [44]. Indeed, in remote Indigenous communities, hygiene and public health interventions, which include handwashing with soap and water, sanitation and hygiene promotion are most likely to reduce diarrhoeal illness, especially in children [45]. In the only outbreak reported in remote Northern Territory, human to human transmission was implicated as the cause of cryptosporidiosis spread in younger age groups. Inadequate provision of drinking water and protection of drinking water sources from faecal contamination has been implicated as the source of several outbreaks of cryptosporidiosis elsewhere [46]. In remote Australia, the lack of adequate quantities of potable drinking water has been identified as a major health issue [47] and may partly be responsible for the high cryptosporidiosis rates in these areas. To develop effective interventions tailored to these settings, we need to target the environmental and social factors and individual behaviors that drive the high rates of disease in remote areas.

Consistent with published studies, strong age related patterns in reported illnesses were identified. The 0–4 year age group had the highest rates of reported cryptosporidiosis consistently over the 12 year period. Such a pattern could be a result of increased contact with sources of infection [48], susceptibility to infection due to immunological naiveté or a greater probability of seeking treatment [49]. As *Cryptosporidium* is transmitted through the faecal-oral route, higher disease rates in adult females between 20 and 39 years may be partly explained by nappy changing and child minding activities (25, 26) which have been identified as risk factors in previous cryptosporidiosis studies [50,51]. In low income settings, some studies have found that cryptosporidiosis in children is associated with malnutrition and was linked to delayed growth and development [3,52,53] and death [4]. While the high rates of cryptosporidiosis in preschool children is widely established [1,22], gaps in our understanding of the factors that drive this high burden of disease remain. A follow up of children with cryptosporidiosis could provide valuable information on immunity to recurrent infections and growth outcomes in remote Australian communities with high cryptosporidiosis rates.

Some studies have found that Indigenous populations and minority ethnic groups have lower rates of reported illnesses and hypothesized that such patterns could be due to poorer access to health resources resulting in lower testing and reporting rates of illnesses [54,55]. However, despite the under reporting associated with surveillance systems [56] and the lack of health resources in these areas [57], we found that that high rates of reported cryptosporidiosis in Australia cluster in areas with a high proportion of ATSI populations (parts of the Northern Territory, which has the highest proportion of ATSI people in Australia, followed by Western Australia). Thus, the real burden of cryptosporidiosis in these communities is likely to be much higher. Cryptosporidiosis is already recognized as an important parasitic disease in the Oceanic region [16] with much higher rates of reported infection in ATSI peoples [57]. Our findings support these studies and add further evidence to show that this is an important issue for public health deserving of ongoing attention with respect to interventions for disease control.

### Study limitations and strengths

The main limitation of this study is that it was based on passive surveillance data, which is known to suffer from significant under reporting [56]. However, the seasonal patterns and marked geographic variation in the rates of reported cryptosporidiosis identified here are indicative of locality specific exposures and risk factors. Such an analysis is only possible with data that are collected across space and over time. There has been no change of reporting practices for *Cryptosporidium* that has occurred over the time period of analysis, either nationally or locally. The incompleteness of ATSI status reporting, particularly in Queensland where only 48% of the data were complete, indicates that detailed statistical analysis using the current ATSI status data would be unwise at the national scale. Finally, different species can be spatially and temporally distinct in their prevalence and transmission pathways, potentially resulting in different patterns of disease incidence [58]. Although we were unable to address species differences, this also represents strength of our study. Molecular methods to identify species and serotypes may differ across States and Territories accounting for inter-state differences in disease patterns, therefore analysis at genus level only is most reliable across Australia and internationally.

### Conclusions

Rates of reported cryptosporidiosis in Australia are higher than many comparable developed countries. Through our analysis of reported illness data and summary of published and health department literature we show that the majority of these studies are short term outbreak reports and focused molecular studies, providing limited understanding of disease patterns Australia-wide.

We identified strong seasonal patterns in notification data that suggest environmental factors are important predictors of cryptosporidiosis risk. Investigating the association between cryptosporidiosis and regional weather variability and proximity to livestock will provide important information on environmental transmission of *Cryptosporidium*.

The finding that children under four years old, particularly male children, are at increased risk, and a trend towards higher rates for females in the 20–39 years age group, is a common pattern among enteric infections. However, cryptosporidiosis infection may be a marker for impaired development and cognition in later life. Longitudinal studies that examine how environmental and social factors affect growth and development of children are needed.

Rates of reported cryptosporidiosis are highest in the warm and ATSI population dominated regions of Australia. Moreover, an increasing risk of reported cryptosporidiosis with remote living is evident. The high rates of reported cryptosporidiosis in remote and ATSI dominated regions suggest that the real incidence of cryptosporidiosis in these communities is potentially much greater. Effective prevention and control of cryptosporidiosis requires interventions and promotions aimed at the environmental, societal and individual exposures that underlie this localization of high cryptosporidiosis risk. Isolated epidemiological investigations conducted in urban areas are unlikely to provide an insight into disease transmission in remote areas. To control cryptosporidiosis in Australia, tailoring effective health interventions/promotion for remote communities need to be a priority for public health research and action.

## Supporting Information

S1 Checklist STROBE checklist. (DOC)

S1 Fig Average age and gender specific rates of reported cryptosporidiosis per 100 000 population across Australian states and territories, 2001–2012. (TIF)

S2 Fig Spatial distributions of climatic regions and remoteness categories using 2011 postal area boundaries. (TIF)

S3 Fig Average rates of reported cryptosporidiosis per 100 000 population for (A) 2001–2005 using 2001 postal area boundaries (B) 2006–2010 using 2006 postal area boundaries (C) 2011–2012 using 2011 postal area boundaries. (TIF)

S1 Table Summary of 41 studies focused on cryptosporidiosis in Australia; study focus, location and design and main findings. (DOCX)
