# Supplementary figures and images for: Cryptosporidiosis: A Disease of Tropical and Remote Areas in Australia
Source: PLoS Negl Trop Dis. 2015 Sep 22;9(9):e0004078. doi: 10.1371/journal.pntd.0004078 (PMC4579119; doi:10.1371/journal.pntd.0004078)

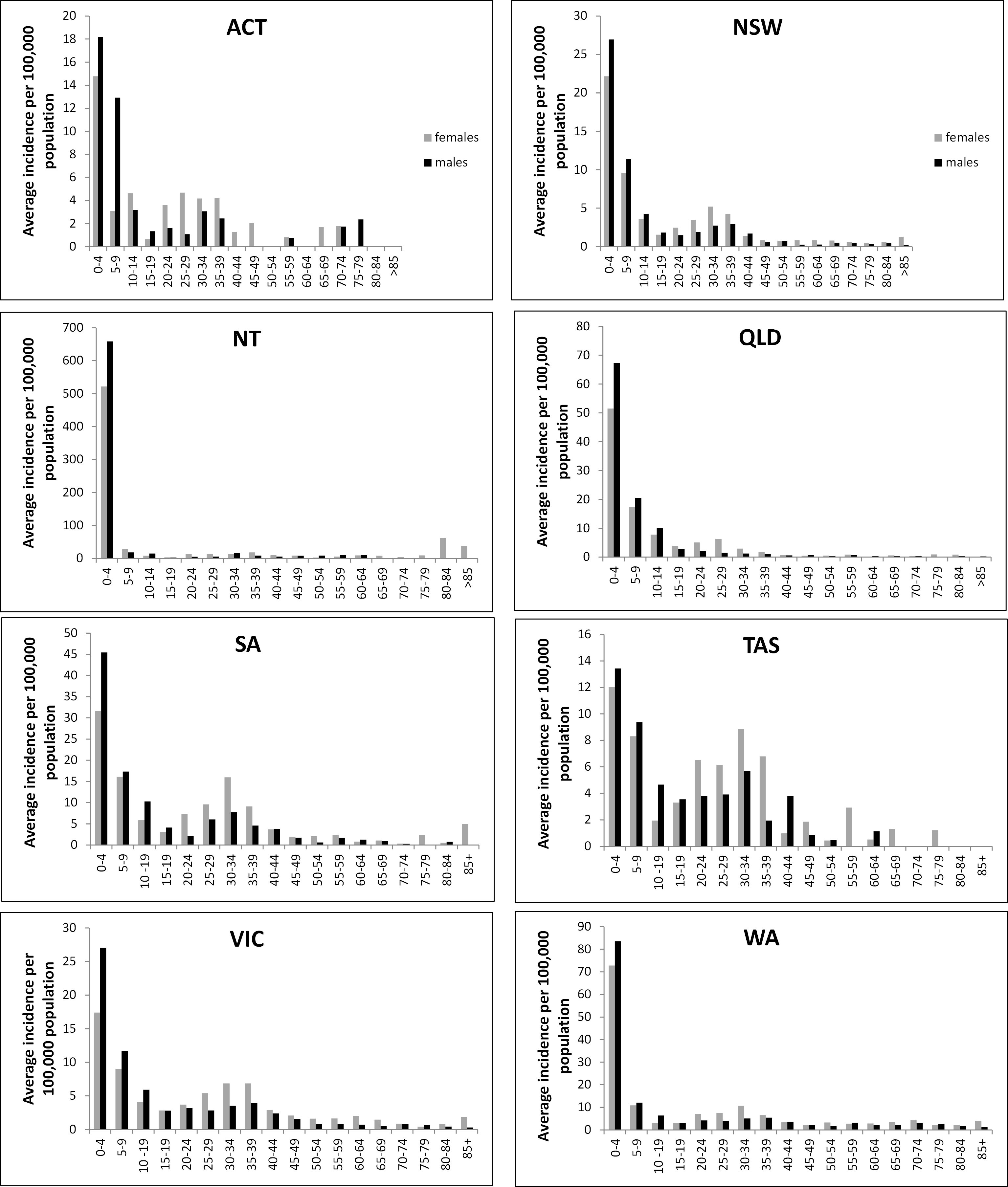

Supplement: S1 Fig — (TIF) [file pntd.0004078.s002.tif]

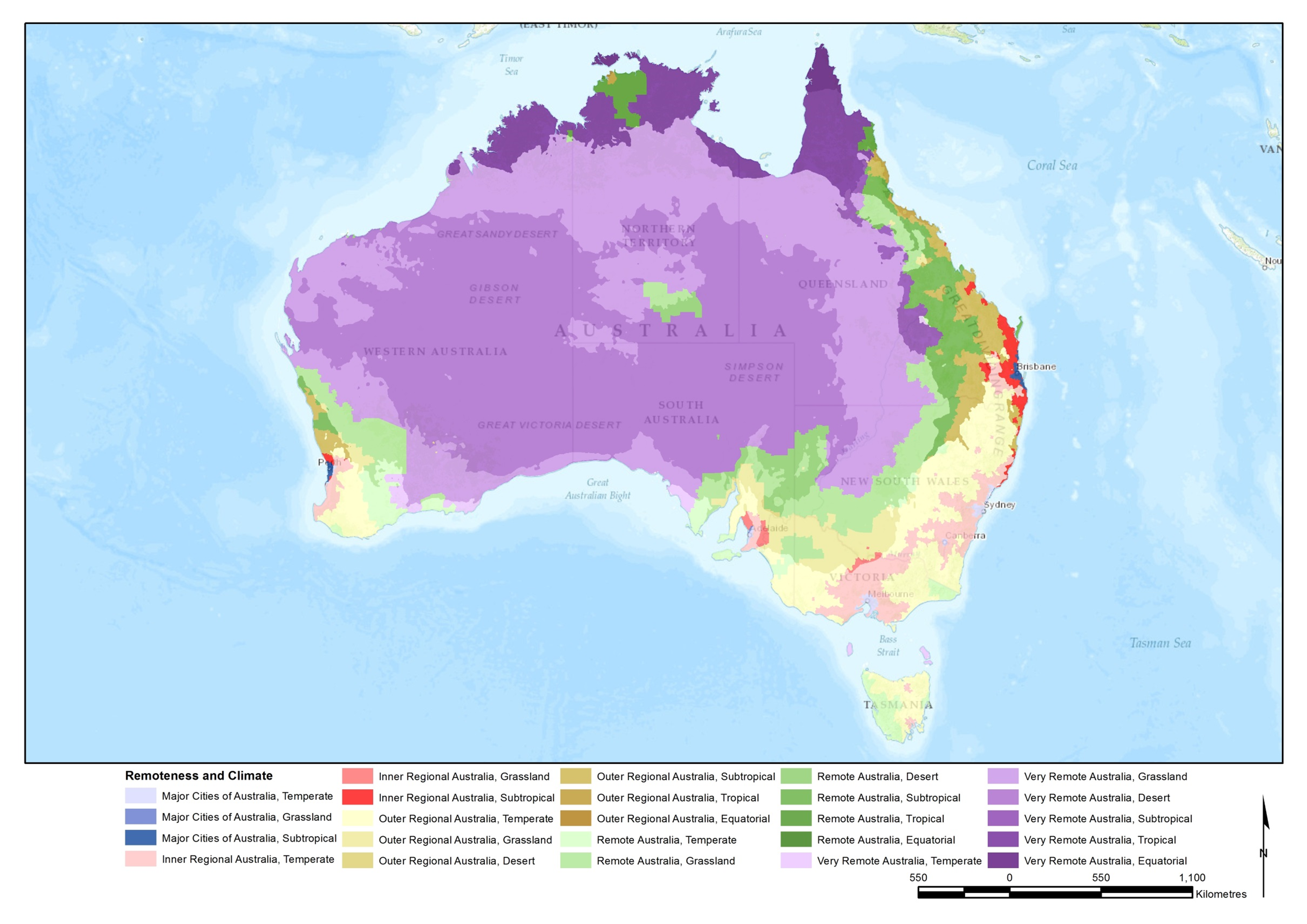

Supplement: S2 Fig — (TIF) [file pntd.0004078.s003.tif]

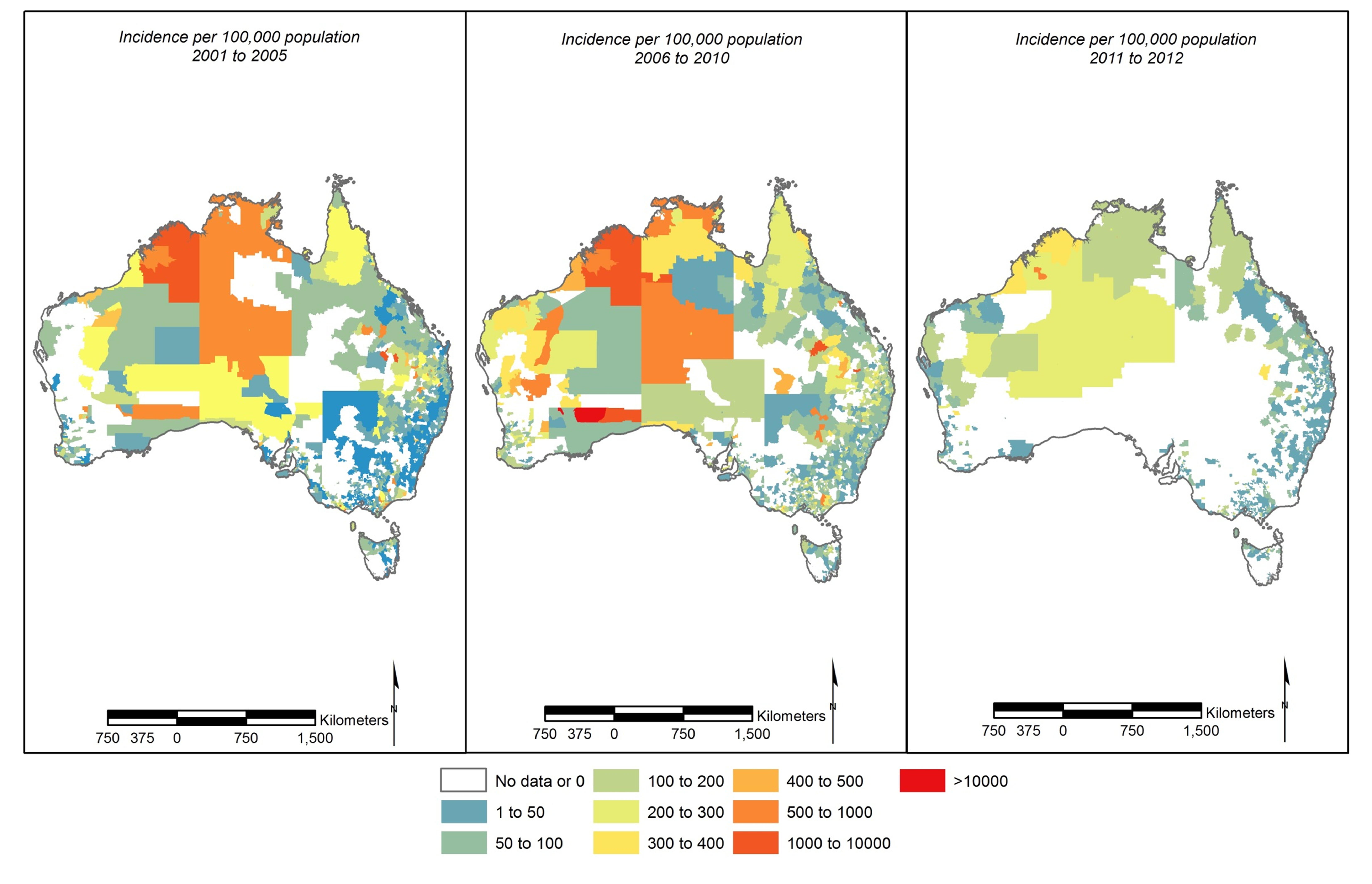

Supplement: S3 Fig — (TIF) [file pntd.0004078.s004.tif]
