# Supplementary material for: Cryptosporidiosis: A Disease of Tropical and Remote Areas in Australia
Source: PLoS Negl Trop Dis. 2015 Sep 22;9(9):e0004078. doi: 10.1371/journal.pntd.0004078 (PMC4579119; doi:10.1371/journal.pntd.0004078)
Supplement: S1 Table — (DOCX) [file pntd.0004078.s005.docx]

Table S1 Summary of 43 studies focused on cryptosporidiosis in Australia; study focus, location and design and main findings

| **Study Design** | **Setting**  **Year (Reference)** | **Location** | **Main findings, including risk factors for epidemiological studies** |
| --- | --- | --- | --- |
| **Case-Control** | Outbreak  2008 ([1](#_ENREF_1)) | Urban  New South Wales | Single point source swimming pool identified as cause of outbreak (31 confirmed cases). Risk factors identified included swimming at facility A and household member with diarrhoea. Animal contact reduced odds of illness. |
|  | Outbreak  1997-1998 ([2](#_ENREF_2)) | State-wide New South Wales | Multiple swimming pool sources identified. Younger mean age of cases (n=100) at 4.2 years versus 7.1 years for controls (n=200). Risk factors identified included swimming at a public pool, swimming in a dam, river or lake, and cases were less likely to drink bottled water. *Cryptosporidium* oocysts detected more commonly in water from case pools (n=6) than control pools (n=2). |
|  | Outbreak  2001 ([3](#_ENREF_3)) | Urban  Queensland | Unpasteurized milk identified as the cause of the outbreak (8 confirmed cases). Six of 10 unpasteurised milk samples positive for *Cryptosporidium* antigen. |
|  | Outbreak  1998 ([4](#_ENREF_4)) | Urban  Victoria | Swimming at pool “X” associated with illness (7 confirmed cases in analysis). Cryptosporidium not detected in any of the four pools at implicated facility sampled. |
|  | Outbreak  1998 ([5](#_ENREF_5)) | Urban  Queensland | Swimming pool complex identified as cause of outbreak (31 confirmed cases in analysis).  Three of four pools at Complex A positive for *Cryptosporidium* oocysts. |
|  | Outbreak  1994-1995 ([6](#_ENREF_6)) | Urban  New South Wales | Swimming in a particular indoor swimming pool associated with illness (43 confirmed cases in analysis). Oocysts were also detected in water from the swimming pool. |
|  | Sporadic  1998-2001([7](#_ENREF_7), [8](#_ENREF_8)) | Urban Victoria & South Australia | Significant risk factors identified by region as:  Victoria, Melbourne (201 cases): swimming in public pools, contact with a child with diarrhoea, household member with diarrhoea and calf contact.  South Australia, Adelaide (134 cases): contact with a child with diarrhoea, household member with diarrhoea, calf contact and drinking unboiled water from a river, lake or dam. |
|  | Sporadic cases  1990-1991([9](#_ENREF_9)) | Urban  South Australia | No significant risk factors identified (51 confirmed cases in analysis), but cases tended more than controls to have consumed only spring water, or only mains water; controls more likely to have consumed only rainwater than cases. |
| **Ecological study** | Sporadic  2001 ([10](#_ENREF_10)) | State-wide Queensland | Temperature and SEIFA were statistically associated with the probability of occurrence of cryptosporidiosis across Queensland |
|  | Sporadic  1996-2004 ([11](#_ENREF_11)) | Urban  Queensland | Infection associated with warmer, drier conditions. |
|  | Sporadic  1996-2004 ([12](#_ENREF_12)) | Urban  Queensland | Cryptosporidiosis risk was best explained by an interaction between SEIFA and the proportion of residents with low educational attainment at an SLA level |
|  | Serological survey  1998 ([13](#_ENREF_13)) | Urban  New South Wales & Victoria | No serological evidence to suggest increased risk of infection in Sydney residents (Sydney drinking water contaminated with high levels of *Cryptosporidium)* when compared to Melbourne residents (Melbourne drinking water supply infrequent detections of Cryptosporidium). |
|  | Survey  2010 ([14](#_ENREF_14)) | Rural  New South Wales | Prevalence in calves: 73.5% (144/196), identified *C. parvum, C. bovis* and *C. ryanae.*  Prevalence in humans: 23.8% (15/63), identified *C. parvum* and *C. bovis.* |
| **Descriptive epidemiology: Case series** | Outbreak  2005 ([15](#_ENREF_15)) | Urban  New South Wales | 38% (11/29) of cases interviewed swam at the same indoor heated pool.  Swimming pool water detected *Cryptosporidium* oocysts. |
|  | Outbreak  2005 ([16](#_ENREF_16)) | Urban  New South Wales | 53% (89/178) reported swimming in a public pool in 12 days before onset of symptoms. Four of five pools tested were positive for *Cryptosporidium* oocysts. |
|  | Outbreak  2001-2002([17](#_ENREF_17), [18](#_ENREF_18)) | Urban  Northern Territory | Cases reported attending childcare centres (36/57, 63%) and swimming pools (18/57, 32%), with some of the cases clustered in one childcare centre (7 cases) and one public pool (7 cases). |
|  | Outbreak  2001 ([19](#_ENREF_19)) | State-wide  Tasmania | 81% (29/36) had attended the same agricultural show, and 75% (27/36) reported contact with animals in the animal nursery. |
|  | Surveillance data analysis  1990-2000([20](#_ENREF_20)) | State-wide New South Wales | Average yearly notification rates were highest in the 0–4 year age group (43.4/100,000 population), followed by 5–9 years (14.8/100,000), geographical and seasonal variation in disease rates, including peak rates in summer. |
|  | Outbreak  2001([21](#_ENREF_21)) | Urban  Northern Territory | Cases clustered in multiple child care centres, no source identified (43 cases investigated). |
|  | Outbreak 1999([22](#_ENREF_22)) | Rural  Northern Territory | Human to human transmission implicated in younger age groups |
|  | Outbreak  1998([23](#_ENREF_23)) | Urban  Victoria | Seven clusters associated with public swimming pools (53 cases across these pools) and one cluster associated with a childcare centre (4 cases). |
|  | Surveillance data analysis  2001-2009([24](#_ENREF_24)) | State-wide  Victoria | Annual notification rates increased in two outbreak years (2006 and 2009) at 20/100 000, compared to 4-12/100 000 in other years; rates highest in children (40.7/100 000 for children less than 10 compared with 6.5/100 000 for those aged 10 years and older); peak of cases in warmer months (February-April); and two-thirds of cases reported form metropolitan regions. |
|  | Hospital data  2007-2010 ([25](#_ENREF_25)) | Urban  New South Wales | *Cryptosporidium* was identified as the causative agent in 7% (18/910) of people hospitalized for enteric protozoan infections in four Sydney hospitals. |
|  | Prevalence survey  1988*([26](#_ENREF_26)) | Urban and remote  Queensland | 36 (4.6%) faecal samples from 780 patients from the Townsville and remote Palm Island region were positive for *Cryptosporidium*. |
|  | Clinical treatment assessment  2007-2012([27](#_ENREF_27)) | Urban and remote  Northern Territory | Nitazoxanide may be an effective treatment for cryptosporidiosis in Indigenous children (n=28). |
|  | Serological survey  1989-1991([28](#_ENREF_28)) | Urban  Victoria | Indication of recent infection in 8% (29/369) of children and 5 % (5/79) of adults. |
| **Descriptive epidemiology:**  **Case report** | Case report  2013*([29](#_ENREF_29)) | Urban and travel  Western Australia | Mixed infection in one person with three species of *Cryptosporidium*; two wildlife associated (*C.* *meleagridis* and *C. mink* genotype) and one unknown species. Case reported interstate (Queensland) and overseas (Papua New Guinea) travel in incubation period, drank untreated water and swam in public swimming pools. (*16 Ng-Hublin 2013)* |
|  | Surveillance report 2007 ([30](#_ENREF_30)) | Urban South West,  Western Australia | Clusters of reported disease associated with animal petting farms and domestic swimming pool. |
|  | Surveillance report  2003([31](#_ENREF_31)) | Northern Territory | Quarterly surveillance report, July – September 2003, reported 1 case in the quarter, considerably below mean (14) for quarter in previous four years |
| **Molecular analysis** | Outbreak  2009([32](#_ENREF_32)) | State-wide  New South Wales | Public swimming pool use contributing factor to outbreak. Anthroponotic *C*. *hominis* IbA10G2 subtype (449 confirmed cases) identified as the causative parasite.  Spatiotemporal analysis identified outbreak confined to densely populated coastal cities of Sydney and Newcastle. |
|  | Outbreaks  2007 ([33](#_ENREF_33)) | Urban (majority)  South Australia & Western Australia | Western Australia (n=48): all isolates were *C. hominis*  South Australia (n=24): 21 *C. hominis* isolates two C*. parvum* isolates and 1 was mixed with both (*C*. *parvum* and *C.* *hominis*) . |
|  | Sporadic  2008-2011([34](#_ENREF_34)) | State-wide  New South Wales | Human infections (n=261) were mixed: with predominantly human and cattle subtypes (*C. hominis 66%, C. parvum 33%, C. andersoni 0.5%* and *C. fayeri 0.5%*.  Cattle samples (n=205) identified as *C. hominis, C. parvum, C. bovis* and *C. ryanae.* |
|  | Sporadic  2008([35](#_ENREF_35)) | Rural  New South Wales | Cattle samples identified *C. parvum* (14) and *C. bovis* (1).  Human infections were mixed with cattle and human subtypes (4 *C. parvum*, 3 *C. hominis*). |
|  | Sporadic  2005-2008([36](#_ENREF_36)) | State-wide  Western Australia | Majority of illnesses due to *C. hominis*  at 78.6%, 19.8% due to *C. parvum* and 1.6% with *C. meleagridis* (of 248 tested). |
|  | Sporadic  2006-2007([37](#_ENREF_37)) | Urban  New South Wales | 58% of cases (19/33) identified as *C. parvum*.  Molecular evidence that human infections were of anthroponotic and zoonotic transmission. |
|  | Sporadic  2009*([38](#_ENREF_38)) | State-wide  New South Wales | Report on genotypes (subtypes) of a selection on human cases (n=69).  Extensive variation was observed within the GP 60 locus for genotyping of human samples (*C. parvum* and *C. hominis*). |
|  | Sporadic  2008*([39](#_ENREF_39)) | State-wide  South Australia | Report on typing of a selection of human cases. *C. hominis* (n= 38) and *C. parvum* (n= 24) were identified. Sequences and subtypes identified. |
|  | Sporadic  2014*([40](#_ENREF_40)) | State-wide  Tasmania | Report on typing of a selection of human cases. *C. hominis* (n=66), *C. parvum* (n=15) and an unidentified genotype (n=1) from traveller to Indonesia identified. |
|  | Sporadic  2008*([41](#_ENREF_41)) | Australia | Report on speciation of a selection of human and cattle cases. 82% of human cases (41/50) infected with *C. hominis* and 18% with *C. parvum*. All cattle samples (n=7) were *C. parvum*. |
|  | Case report  2009 ([42](#_ENREF_42)) | Rural  New South Wales | *C. fayeri* identified in human case with same subtype as previously identified in a kangaroo. |
|  | Case report  2014*([43](#_ENREF_43)) | Unspecified  Victoria | Genetic analysis of human isolate. New *C. cuniculus* genotype identified in human, similar to genotype identified in a kangaroo. |

*Indicates publication date as no study period dates provided in publication.

1. Mayne DJ*, et al.* (2011) A community outbreak of cryptosporidiosis in sydney associated with a public swimming facility: a case-control study. *Interdisciplinary perspectives on infectious diseases* 2011:341065.

2. Puech M*, et al.* (2001) A statewide outbreak of cryptosporidiosis in New South Wales associated with swimming at public pools. *Epidemiology and infection* 126(03):389-396.

3. Harper CM, Cowell NA, Adams BC, Langley AJ, & Wohlsen TD (2002) Outbreak of *Cryptosporidium* linked to drinking unpasteurised milk. (Translated from eng) *Communicable diseases intelligence quarterly report.* 26(3):449-450 (in eng).

4. Hellard M*, et al.* (2000) An outbreak of cryptosporidiosis in an urban swimming pool: why are such outbreaks difficult to detect? *Australian and New Zealand journal of public health* 24(3):272-275.

5. Stafford R, Neville G, Towner C, & McCall B (2000) A community outbreak of Cryptosporidium infection associated with a swimming pool complex. *Communicable diseases intelligence.* 24(8). <http://www.health.gov.au/internet/main/publishing.nsf/Content/cda-pubs-cdi-2000-cdi2408-cdi2408c.htm>.

6. Lemmon J, McAnulty J, & Bawden-Smith J (1996) Outbreak of cryptosporidiosis linked to an indoor swimming pool. *Medical Journal of Australia* 165(11-12):613-616.

7. Robertson B*, et al.* (2002) Case-control studies of sporadic cryptosporidiosis in Melbourne and Adelaide, Australia. *Epidemiology and Infection* 128(3):419-431.

8. Robertson B*, et al.* (2001) Case-Control studies of sporadic Cryptosporidiosis in Melbourne and Adelaide. Victorian Infectious Diseases Bulletin. 4.4.

9. Weinstein P, Macaitis M, Walker C, & Cameron S (1993) Cryptosporidial diarrhoea in South Australia. An exploratory case-control study of risk factors for transmission. *The Medical journal of Australia* 158(2):117-119.

10. Hu W, Mengersen K, & Tong S (2010) Risk factor analysis and spatiotemporal CART model of cryptosporidiosis in Queensland, Australia. *BMC infectious diseases* 10(1):311.

11. Hu W, Mengersen K, Fu S-Y, & Tong S (2010) The use of ZIP and CART to model cryptosporidiosis in relation to climatic variables. (Translated from English) *Int J Biometeorol* 54(4):433-440 (in English).

12. Hu W, Mengersen K, & Tong S (2009) Spatial analysis of notified cryptosporidiosis infections in Brisbane, Australia. (Translated from eng) *Annals of epidemiology* 19(12):900-907 (in eng).

13. Frost FJ*, et al.* (2000) Serological evaluation of Cryptosporidium oocyst findings in the water supply for Sydney, Australia. *International Journal of Environmental Health Research* 10(1):35-40.

14. Ng JS*, et al.* (2012) Evidence of Cryptosporidium transmission between cattle and humans in northern New South Wales. *Experimental parasitology* 130(4):437-441.

15. Paterson J & Goldthorpe I (2006) Managing a cluster of cryptosporidiosis associated with a public swimming pool. *New South Wales public health bulletin* 17(6):80-80.

16. Black M & McAnulty J (2005) The investigation of an outbreak of cryptosporidiosis in New South Wales in 2005. *New South Wales public health bulletin* 17(6):76-79.

17. Markey P (2002) Cryptosporidiosis: will it happen this wet season? : The Northern Territory Disease Control Bulletin. 9.4. Centre for Disease Control, Department of Health.

18. Markey P (2002) Cryptosporidiosis outbreak in Darwin and Palmerston. The Northern Territory Disease Control Bulletin. 9.1. Centre for Disease Control, Department of Health.

19. Ashbolt R, Coleman D, Misrachi A, Conti J, & Kirk M (2003) An outbreak of cryptosporidiosis associated with an animal nursery at a regional fair. *Communicable diseases intelligence.* 27(2):244-249.

20. Menzies R (2002) Cryptosporidiosis in NSW 1990-2000. *NSW Public Health Bulletin* 13:54-57.

21. Peacock D (2001) Darwin cryptosporidiosis outbreak December 2000 - January 2001. The Northern Territory Disease Control Bulletin. 8.1. Centre for Disease Control, Department of Health.

22. Dentith H & Alexander I (1999) Cryptosporidium parvum: An associated outbreak of diarrhoeal disease in Nhulunbuy, East Arnhem. The Northern Territory Disease Control Bulletin. 6.4. Centre for Disease Control, Department of Health.

23. Kirk M, Andrews R, Tallis G, Densten K, & Hellard M (1998) Clusters of Cryptosporidium Infection in Victoria. Victorian Infectious Diseases Bulletin. 1.1. Victorian Department of Health.

24. Kent L, Higgins N, & McPherson M (2011) The epidemiology of cryptosporidiosis in Victoria, 2001–2009. Victorian Infectious Diseases Bulletin. 14.2. Victorian Department of Health.

25. Fletcher S*, et al.* (2014) Epidemiology and Geographical Distribution of Enteric Protozoan Infections in Sydney, Australia. *Journal of Public Health Research* 3(2):298.

26. Cruickshank R, Ashdown L, & Croese J (1988) Human cryptosporidiosis in North Queensland. (Translated from eng) *Aust N Z J Med* 18(4):582-586 (in eng).

27. McLeod C, Morris P, Snelling T, Carapetis J, & Bowen A (2014) Nitazoxanide for the treatment of infectious diarrhoea in the Northern Territory, Australia 2007-2012. *Rural and remote health* 14(2759):1.4-7.3.

28. Groves VJ, Lehmann D, & Gilbert GL (1994) Seroepidemiology of cryptosporidiosis in children in Papua New Guinea and Australia. *Epidemiol Infect* 113(3):491-499.

29. Ng-Hublin JS, Combs B, Mackenzie B, & Ryan U (2013) Human cryptosporidiosis diagnosed in Western Australia: a mixed infection with Cryptosporidium meleagridis, the Cryptosporidium mink genotype, and an unknown Cryptosporidium species. *Journal of clinical microbiology* 51(7):2463-2465.

30. Western Australian Communicable Disease Control Directorate (2007) Cryptosporidiosis Alert. Disease WAtch: the Western Australian Communicable Diseases Bulletin. 11.1. Western Australian Department of Health.

31. Dempsey K (2003) Enteric diseases in the Northern Territory July - September 2003. The Northern Territory Disease Control Bulletin. 10.4. Centre for Disease Control, Department of Health.

32. Waldron LS*, et al.* (2011) Molecular epidemiology and spatial distribution of a waterborne cryptosporidiosis outbreak in Australia. *Applied and Environmental Microbiology* 77(21):7766-7771.

33. Ng JS, Pingault N, Gibbs R, Koehler A, & Ryan U (2010) Molecular characterisation of Cryptosporidium outbreaks in Western and South Australia. *Experimental parasitology* 125(4):325-328.

34. Waldron LS, Dimeski B, Beggs PJ, Ferrari BC, & Power ML (2011) Molecular epidemiology, spatiotemporal analysis, and ecology of sporadic human cryptosporidiosis in Australia. *Appl Environ Microbiol* 77(21):7757-7765.

35. Ng J*, et al.* (2008) Evidence supporting zoonotic transmission of *Cryptosporidium* in rural New South Wales. *Experimental parasitology* 119(1):192-195.

36. Ng J, MacKenzie B, & Ryan U (2010) Longitudinal multi-locus molecular characterisation of sporadic Australian human clinical cases of cryptosporidiosis from 2005 to 2008. *Experimental parasitology* 125(4):348-356.

37. Alagappan A*, et al.* (2008) Development of fluorescent in situ hybridization for Cryptosporidium detection reveals zoonotic and anthroponotic transmission of sporadic cryptosporidiosis in Sydney. *Journal of microbiological methods* 75(3):535-539.

38. Waldron LS, Ferrari BC, & Power ML (2009) Glycoprotein 60 diversity in C. hominis and C. parvum causing human cryptosporidiosis in NSW, Australia. *Experimental parasitology* 122(2):124-127.

39. Jex AR*, et al.* (2008) Classification of Cryptosporidium species from patients with sporadic cryptosporidiosis by use of sequence-based multilocus analysis following mutation scanning. *Journal of clinical microbiology* 46(7):2252-2262.

40. Koehler AV*, et al.* (2014) First genetic analysis of Cryptosporidium from humans from Tasmania, and identification of a new genotype from a traveller to Bali. *Electrophoresis*.

41. O'Brien E, McInnes L, & Ryan U (2008) Cryptosporidium GP60 genotypes from humans and domesticated animals in Australia, North America and Europe. *Experimental parasitology* 118(1):118-121.

42. Waldron LS, Cheung-Kwok-Sang C, & Power ML (2010) Wildlife-associated Cryptosporidium fayeri in human, Australia. *Emerging infectious diseases* 16(12):2006-2007.

43. Koehler AV, Whipp MJ, Haydon SR, & Gasser RB (2014) Cryptosporidium cuniculus - new records in human and kangaroo in Australia. *Parasites & vectors* 7:492.
